# Cost of provision of essential health Services in Public Health Centers of Jimma zone, Southwest Ethiopia; a provider perspective, the pointer for major area of public expenditure

**DOI:** 10.1186/s13561-021-00334-y

**Published:** 2021-09-13

**Authors:** Diriba Feyisa, Kiddus Yitbarek, Teferi Daba

**Affiliations:** 1 Department of Pharmaceutics and Social Pharmacy, School of Pharmacy, College of Health Sciences, Mizan -Tepi University, Mizan-Aman, Ethiopia; 2 Department of Health Economics, Policy and Health Services Management, College of Public Health, Institute of Health, Jimma University, Jimma, Ethiopia

**Keywords:** Cost, Essential health services, Public health centers, Jimma, South West Ethiopia

## Abstract

**Background:**

Provision of up-to-date cost information is crucial for not only addressing knowledge gap on the cost of essential health services (EHS) but also budgeting, allocating adequate resources and improving institutional efficiency at public health centers where basic health services are delivered the most.

**Objective:**

To analyze the costs of essential health services at public health centers in Jimma Zone.

**Methods:**

A facility based cross-sectional study was conducted in public health centers of Jimma zone from April 10, 2018 to May 9, 2018. The study was conducted from a provider perspective using retrospective standard costing approach of one fiscal year time horizon. Step-down allocation was used to allocate costs to final services. All costs for provision EHS were taken into account and expressed in United States dollar (USD). Sixteen public health centers located in eight districts were randomly selected for the study.

**Results:**

The Average annual cost of providing essential health services at health centers in Jimma zone was USD 109,806.03 ± 50,564.9. Most (83.7%) of the total Annual cost was spent on recurrent items. Nearly half (45%) of total annual cost was incurred by personnel followed by drugs and consumables that accounted around one third (29%) of the total Annual cost. Around two third (65.9%) of the total annual cost was incurred for provision of EHS at the final cost center. The average overall unit cost was USD 7.4 per EHS per year.

**Conclusion:**

Cost providing an EHS at public health centers was low and so, necessitating funding of significant resources to provide standard health care. The variability in unit costs and cost components for EHS also suggest that the potential exists to be more efficient via better use of both human and material resources.

**Supplementary Information:**

The online version contains supplementary material available at 10.1186/s13561-021-00334-y.

## Background

The aim of Universal Health Coverage (UHC) as per the World Health Organization (WHO) is to ensure that all individuals receive the quality health services they require without facing financial difficulties. However, delivering essential health services at a reasonable cost that realizes the ultimate goal of Universal Health Coverage (UHC) is a worldwide challenge [1]. EHS is a package, limited list of services and policy interventions that are cost-effective, equitable, efficient, affordable, and acceptable designed to direct resources to priority areas of health service delivery to reduce disease burdens and ensure equity in health [2]. Essential Health services are considered an integral part of UHC and the provision of good quality health care. EHS is the priority for sustainable development goals (SDGs) as a path to achieve UHC goals [2, 3]. The global interest in EHS also came with the resolution adopted on Universal Health Coverage (UHC) by the 58th World Health Assembly in 2005 [4].

Ethiopia designed its EHSP (Essential health services package) in 2005 to commit scarce resources to the services that provide the best value for money. The aspiration to have public sector facilities that provide a minimum standard of care and fosters an integrated service delivery approach is crucial for advancing population health [5]. Thus, based on the EHSP, the essential health service package is defined as basic health services that the government can avail and provide to treat and control those health problems that are more prevalent in an area, and thereby serve citizens in an equitable manner [6]. In the light of this, the Federal Ministry of Health (FMOH) advocates provisions of EHS and reassuring its commitment to ensuring the provision of good quality EHS at the primary care level. The Health Sector Transformation Plan (HSTP 2015–2035) in the title, **‘***Envisioning Ethiopia’s Path to Universal Health Care through strengthening of Primary HealthCare’* highlights the Ethiopian governments’ commitment to universal health coverage [7].

The costs of essential health services are determined by identifying all resources used in its production and all resources spent by health centers to provide the actual annual essential health services during the fiscal year. They are distinguished as recurrent or capital costs. Recurrent costs are those incurred in the course of a year and are usually purchased regularly includes salary and benefits, drug & consumables, and administration & utilities. Capital cost refers to those that last longer than 1 year, such as buildings, vehicles, equipment, and furniture [8].

Ethiopian health care is mainly funded by the government and donor funds and maimed by financial constraints or inadequate funding limiting the development of the health sector. Health managers are ignorant about the actual cost of the services they produce and deliver. The very notion of “cost” is not clear to the great majority of them [9]. In addition to this, the actual costs of components of health care delivery system are also often not available as budgetary provisions cover only the recurrent expenditures like salary and drugs but do not reflect the investments already done to the infrastructure and equipment [9–11].

Cost data becomes vital in facilitating adequate financing and effective delivery of essential healthcare services requiring an assessment gap between the total amount of resources needed and the amount available to healthcare facilities. Health centers are the farthest-flung units of health care delivery next to health care posts and tasked with essential health services delivery under the strain of rising healthcare costs. Therefore, it is also crucial to figure out how much the government spends per unit of health care services delivered at health centers [10, 11].

Ethiopia is focusing on providing universal access to quality health care without financial burden and ensuring an adaptive health system to meet the population’s changing health needs [12]. This is also the case in Jimma zone. The majority (89%) of Jimma zone dwellers are rural, with estimated 60 to 80% health problems attributable to preventable and treatable infectious diseases and nutritional problems such as malaria, maternal and child problems, diarrhea, HIV/AIDS, and other diseases and malnutrition. The provision of essential health services through social health protection scheme was implemented in almost half of districts of Jimma zone. Akin to other health facilities in Ethiopia, public health centers of Jimma zone operate under a constraint resource that limits universal coverage of essential health care to its population [13]. Therefore, the effective delivery of essential health services in public health centers of Jimma zone at the best value of money could improve the health care needs of larger segments of the population.

Numerous costing studies have been conducted regarding essential health service provision at health service delivery points in developing countries. However, some of them were conducted over a decade ago [14–26], other studies did not explore health centers [15, 18], some focused on measuring efficiency at service delivery points [14, 18, 26], and few others only focus on cost accounting [27–30]. Besides, a little is known about the costs of providing essential health services at public health centres from provider perspective. This knowledge can better enable health center managers and policy makers to budget and allocate the appropriate resources that will ensure higher quality of health care services at service delivery point. Hence, the current study is done with the aim to fill this crucial gap by analyzing the costs of the essential health services in terms of recurrent, capital and unit cost of providing those essential health services from perspectives of public health centers of Jimma Zone.

## Methods

### Description of the study area and period

The study was conducted in public health centers of Jimma Zone. The Zone is located in Oromiya Regional State, 345 km to the Southwestern direction of the capital of Ethiopia, Addis Ababa. It has 21 Woredas’ (districts) with 555 total Kebeles’ (the smallest administrative units). The zone has 5 public hospitals, 118 health centers, and 555 health posts. The study was carried out from April10, 2018 to May 9, 2018.

### Study design

The study utilized a cross-sectional design from the health provider perspective using step-down costing approach for 2009 EFY (July 8, 2016 – July 31, 2017).

### Sample size and sampling technique

The sample size was determined by using information obtained from WHO Tools for Assessing the Operationally of District Health Systems which suggests that it would be enough to take 40% of the total districts provided that the number of district is greater than 20. As such eight (8) districts were selected [31]. Stratified sampling method with proportional allocation was used for the selection of the districts. Two health centers per district were selected to determine the number of public health centers in the final sample. Accordingly, a total of sixteen public health centers were selected. Firstly, all the 21 districts in Jimma zone were grouped by three strata of districts (Level A, Level B and Level C) where the level of districts given by the zonal health department as per their performances was used as stratum. Secondly, 8 districts (1 district from level A, 2 districts From Level B, and 5 districts from level C) were proportionally determined and then randomly selected. Thirdly, 2-PHC; one health center from district capital and other from rural part of district were selected from the selected districts accounting for districts’ urban-rural disparities. The health centers selected for this study are typical of the country’s primary health care facilities in terms of infrastructure, equipment, staffing, population coverage and mode of operation. Per the guidelines, public health centers on the other hand usually serve a community with a population of 25,000 people and are supposed to provide outpatient and normal child delivery services. HCs are generally headed by the health officer and staffed with program heads in the areas of pharmacy, midwifery, laboratory services, public health, environment and nutrition **(**Additional file 1: Supplementary file Annex I)**.**

### Data collection, tools and quality assurance

Three data collectors were carried out data collection under supervision of the principal investigators by using document review checklist comprised of nine sections: building, equipment, vehicle, human resources/personnel, existing incentives, supplies, operations and maintenance costs among others after one full day training on the study design, costing theory and concepts, data collection methods, data techniques and the data collection instruments. All essential health services costs collected in the data covered a one- year period (2009 EFY) through document review, observation and physical counting of rooms of buildings, and physical inventory of equipment. The prices of items were obtained from store keeper invoices of respective health centers. The cost data regarding personnel, administrative and utilities, medicine, laboratory reagent and consumables supplies, building, equipment, furniture and vehicles were collected by adhering to method forwarded for an economic evaluation of health care programmes [32].

#### Personnel cost

The human resources (HR) module was used to account for personnel costs using the number of health care workers’ and administrative and supportive non–health care workers’ (e.g. accountants, cashier) salaries, benefits, and incentives as inputs. Personnel costs for providing the essential health services were collected health centers payroll record review of all salary, overtime payments and staff benefits based on the most up-to-date data from the Human Resource Department of respective health centers and federal ministry of health.

#### Administrative and utilities cost

The administrative cost comprised of cost on cleaning products, repairs, fuel, lubricants, printing, photocopying, stationary etc. The value of administrative and utility items consumed by health centers were determined through desk reference review of administrative records of monthly payments made to utility companies, cost of stationary and consumable for health center administration, payments made to contractors for maintenance buildings and equipment.

Medicine, laboratory reagents and consumable supplies costs:- The medicines, commodities, and consumable supplies required to deliver the essential health services at study health center were identified, quantified, and multiplied by the unit price of the items. The quantity of various medicines, laboratory reagents, medical supplies and consumables consumed within the reference period were obtained from health centers through desk review and the unit price information were obtained from Review of supply records, stock voucher and invoice kept by pharmacy stores at health centers. Facility surveys were undertaken to assess the capital and physical infrastructure such as building, space, furniture and other equipments present in the health centers.

#### Equipment cost

This included cost of general equipment in the various rooms (waiting room, consulting room etc.) such as fetoscope, thermometer, weighing scales, etc. Numbers of equipment in the inventory were identified through physical inventory. The unit prices of items were obtained from Ethiopian Pharmaceutical Supply Agency (EPSA) Jimma hub through documentary review of capital medical equipment 2017/18 Estimated Price list. Annual depreciation value (using replacement to maintenance cost) was determined to come up with the cost of equipment at health centers.

#### Building cost

The infrastructure details were ascertained room wise along with the purpose for which they were being used. Data were collected through record review and observation of the number of blocks of building and rooms of building that provide essential health services. Unit valuation was done based on annual depreciation value (using replacement cost and maintenance costs) based on review of administrative records.

#### Vehicle costs

This included cost of all functional means of transport such as cars and motorbikes in the health centers that are used for supervision, outreach, and administrative activities. Review of administrative records of health centers was used to collect actual purchase cost and unit purchase price was also obtained from market sources.

#### Furniture costs

Numbers of furniture in the inventory were identified through physical inventory. Review of administrative records at health centers was used to collect cost of furniture. The unit prices of locally obtained equipment such a benches, tables, chairs etc. were obtained from market sources.

Utilization data, such as number of essential health service visit; for instance, number of ANC visits and the number of deliveries at the various health centers, were also collected via document reviews of health centers’ attendance and service utilization registration.

### Cost estimation, allocation and analysis

The cost analysis was conducted from a provider perspective. The study included a representative selection of Health Centers in Jimma Zone. The broad components of services were defined with specifically defined services and activities for implementation at health centers **[**Additional file 1: Supplementary file Annex II**].** The analysis also included all recurrent costs (personnel, drugs, medical supplies, utilities, and other recurrent costs) and all relevant capital costs related to provision each essential health services at each health center [Additional file 1: Supplementary file Annex III]. Several tools were used to measure, classify and estimate essential health services at public health facilities in Jimma zone [Additional file 1: Supplementary file Annex IV and VII]. While there are several methods in costing health care services, WHO, Alliance for Health Policy [4] and a systematic review of costing methods [33], concluded that no method is better than the others in all criteria and the choice of method depends on available data, the study setting and other factors.

Therefore, the step-down allocation (SDA) approach as discussed in the WHO manual was found to be the choice and hence, used in this study [29]. Accordingly, costs for each of the sixteen public health centers were calculated using SDA costing approach that requires the calculation and allocation of both capital and recurrent costs following all main steps; (1) defining the final product, (2) defining cost centres, (3) identifying the full cost for each input, (4) assigning inputs to cost centres, and (5) allocating all costs to final cost centres. Firstly, The final product were defined as curative care service, chronic care service,emergency care service, maternal health service (ANC, PNC, Institutional delivery), family planning services, child health services and EPI services. Secondly, cost centers based on the functions of the departments were defined. Three cost centers were identified: overhead, intermediate and final costs centers. Thirdly, cost for each input was identified. A list of resources generated and grouped into the following cost categories:-personnel, administration, drugs & medical supplies, building and equipment costs. The total annual costs were estimated by multiplying and summing quantities consumed on each specific item by the unit price.

Fourthly, costs were assigned to the three cost centers. Some costs can be assigned immediately to certain cost centers. Accordingly, personnel, administration, pharmacy, laboratory and vehicle costs were assigned directly to the relevant cost centers. In addition, personnel, building cost, equipment was distributed to direct cost centers. Fifthly, all costs reallocated from the two cost centers (indirect and intermediate) cost to the direct cost centers. In the final step, the unit cost was calculated. The allocated costs for each direct cost center was divided by the number of visits of each of these centers. Accordingly, the unit cost for direct services was calculated [Additional file 1: Supplementary file Annex V].

All cost were collected in local currency which is Ethiopian birr (ETB) and results presented in USD using 2017 average interbank exchange rate of 22.4 ETB to 1 US$. Total, average and unit costs were estimated [34]. Annual costs were calculated for each of the each health center using a full costing approach involving calculation of both capital and recurrent costs. The cost components included in the analysis were: personnel cost, administrative cost, medicine and consumables cost (representing recurrent cost), cost of equipment, buildings, vehicles and furniture (representing capital cost). Personnel cost was estimated through taking salaries of the staff and by adding the duty incentives as a benefit. Costs related to medical supplies were estimated by taking the total quantity of the supplies at each department and using the current market cost of the supplies and multiplying together [1]. The administration costs which included maintenance, water and electricity, was estimated by taking 5% of medical equipment for maintenance using floor area/building room/ for water electricity and cleaning. Vehicle and motor bike costs were also be estimated in the same way as for medical equipments [29].

Capital costs were annualized to allow for differential timing of capital costs. Thus it gives the equivalent annual cost of the capital cost by spreading the capital costs over their useful life years [16]. In this study, capital costs were annualized using a discount rate with their respective useful life years. Capital costs were annualized using a discount rate with their respective useful life years. A discount rate of 5% was chosen in conformity with most economic evaluation studies conducted in developed and developing countries and in the absence of any accepted alternative rate used in Ethiopia. Based on expert opinion and literature review a useful life of 10 years was used for equipment and 30 years for building [29].

Total cost for running each health center in a year was calculated by adding the annual cost on personnel, administrative, medicine, consumables, equipment and vehicles. The average cost of running a health center was obtained by summing the cost of running all the health centers divided by the number of health centers.

### Data processing and analysis

Data was edited, coded, entered and analyzed using SPSS version 20 and Microsoft Office Excel 2007. Descriptive analysis was applied to display frequency, percentage, total cost, and unit costs health services. Unit costs of each service was calculated by dividing the costs of inputs incurred along each of the services during the base year by the total number of output of the respective services of that year.

### Sensitivity analysis

The Univariate sensitivity and threshold analysis were performed to see the effect of changes of variables that are subject to change over time to assist in the generalization of the study results. Given that several studies use 3–7% discount rates, to assess the impact of using a higher discount rate (7%) was used. The life span of buildings was also varied (from 30 years to 20 years). In addition, with expectation of a future increase in utilization of EHS at the health centers, a 10% increase was assumed and then, the threshold for significance was set at 10% change in costs or higher [29, 32, 33]. The base value of salaries, price of capital items were varied by 25% on both sides. Prices of drugs and consumables show wide variation; hence we varied these by 90% on lower limit to 100% on upper limit. We also estimated the sensitivity of the annual cost and unit cost for providing overall services to variations in discount rates i.e. at 7 and 10% respectively.

### Definition of terms and operational definitions of the study

#### Building cost

Cost obtained after total depreciated construction cost of health centers divided to the number of room at health centers.

#### Capital cost

Capital costs are items with lifespan greater than 1 year and are therefore incurred only every few years rather than annually [10, 15]. All resources spent, allocated or due spend on buildings/rooms of buildings, equipments, Furniture’s and Vehicles purchasing at health centers.

#### Recurrent cost

Recurrent costs are items that are used up during a year and are usually purchased regularly. All resources spent, allocated or due spend on personnel, administrative and supportive departments, pharmacy unit/medicines, medical supplies and consumables’ and Laboratory supplies, reagents and consumables’ at health centers.

#### Cost center

These are units/case team/ within the health center that are the centers of activities and will be assigned different categories. They are classified into overhead, intermediate and final cost centers.

#### Overhead cost center

These cost centers provide support services that are necessary for effective running of the health centers. They include likes of administrative, supportive departments and transport.

#### Intermediate cost center

These centers provide ancillary services and support the final cost center. The dealing with patients at these centers is not intensive. They include pharmacy and laboratory.

#### Final cost center/ patient care

These cost centers are directly responsible for patient care and services. They include; out-patient department, child health services, chronic care services, maternal and family health services etc.

#### EFY (Ethiopian fiscal year)

In the Ethiopian calendar, the year starts on September 11 and ends on September 10 according to Gregorian calendar. Hence, EFY 2009 started on July 8, 2016, and ended on July 7, 2017 [35].

#### Per capita expenditure

In this study, per capital expenditure was computed using population of Jimma zone as the base for computation.

### Data quality assurance

The data collection tool was pre-tested. The Principal investigators has supervised the initial procedures including training of data collectors, pre-test and the 1st data collection on 5% of the study health centers, discussed with the data collectors on regular basis and reviewed the collected data for completeness. The collected data were summarized on the same day of the data collection. The quality of the data was confirmed by using different types of documents containing the same information. Entire data were checked by the principal investigator for the consistency, regularity and completeness.

### Ethical consideration

Ethical approval was obtained from the Ethics Review Board of Institute of Health, Jimma University and given to the Oromia Regional Health Bureau (ORHB). Then a letter of cooperation from RHBs was also taken for Jimma Zone. Verbal consent was obtained from health centers’ heads and all other respondents to be contacted for information before enrolling them as the study participant/unit. During the consent process, the respondents got information regarding the purpose of the study, why and how they are selected as the respondents of the study, and what will be expected from them.

## Results

### Background characteristics of health centers

All the selected health centers’ have similar structure having about 19 ± 2.75 rooms. The average number of staff per HCs was 21 ± 5.94. The average actual population covered by a Public Health Centre (PHCs) was 14,579 ± 7992.79. The number of ANC visits, deliveries and PNC visits, immunization service, chronic, curative child health varied among the health centers. **[**Table 1**].**

**Table 1:** The background characteristics at public health centers in Jimma zone for EFY 2009, October 2018

Health center Characteristics (*N* = 16)	PHC (***n*** = 16)		
	Mean	Range [MIN-MAX]	SD
**Number of rooms at Health center**	19	8 (14–22)	2.75
**Population covered/served per year**	14,579	56,466 (19227–75,693)	7992.79
**Human Resources/personnel/**	21	26,214 (6566–32,780)	5.94
**Emergency per year**	1396	18 (11–29)	484.57
**Curative per year**	985	2365 (614–2979)	794.45
**Chronic care per year**	3490	4136 (1077–5213)	604.5
**Maternal (ANC,PNC,delivery, FHS)**	6071	5908 (1539–7447)	323
**ANC**	1821	6717 (1685–8402)	746
**PNC**	849	3313 (820–4133)	349
**Delivery**	972	1547 (382–1929)	398
**Family planning service**	74	1767 (437–2205)	8.25
**Child health per year**	1518	89 (46–135)	69.85
**EPI per year**	1453	1657 (410–2067)	654.4
**Outreach service per year**	985	2762 (683–3445)	565.7

### Total annual costs at health centers

**Fig. 1:**
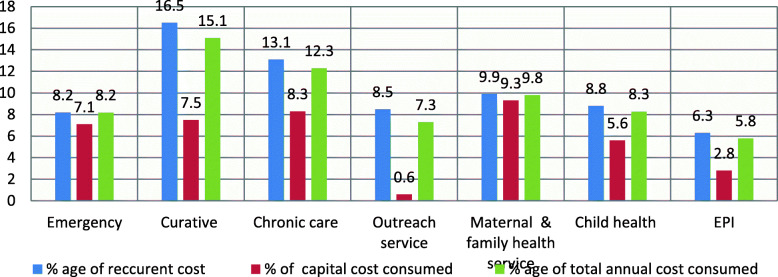
Percentage of total annual cost spent on final cost center of health centers of Jimma zone for EFY 2009, October 2018

The total cost of running the health centers in providing essential health services in the sixteen public health centers in 2009 Ethiopian financial year was USD 1,754,150.18 while the mean annual costs those services at HCs were USD 109,806.03 ± 50,564.9. The largest part of total annual cost USD1,227,872.7 (65.9%)] were absorbed by the final cost centers (the service departments) followed by overhead cost center which absorbed 21.9% of the total annual cost while the intermediate cost center were absorbed only 12.2% percent of total annual expenditure **[**Table 2**].** Two third (65.9%) of the total cost incurred for provision of essential health services at HC was on accounted for various essential health services at the final cost center. Curative health services accounts the highest with 15.2%, followed by chronic care service with 12.5% while EPI consumed the least, with 5.8% of total annual cost per health center **[**Figure 1**].**

**Table 2:** Summary of cost of providing essential health services per cost center (USD) in Jimma zone for EFY 2009, October 2018

cost center	Personnel	Medicine/drugs	Laboratory supplies	Medical consumables	Admn. & utility cost	Building	Equipments	Furniture	Vehicles	total Annual cost
**Overhead**										
Administrative. & utilities	178,654.9					7429.1	5253.1	5373.1		215,019.2
Transport	37,944.4				18,308.9				121,104.9	194,309.9
**Intermediate**					35,260.6					
Pharmacy	75,888.8					7429.1	1500.9	1343.3		104,470.9
Laboratory	75,888.8			1115.6	18,308.9	3525.7	30,018.9	1343.2		120,581.2
**Final**					8688.9					
Emergency	77,469.8	26,463	5385.2	929.7		7429.1	9005.8	2686.5		147,677.8
Curative	77,469.8	120,028.6	34,619.2	8075.1	18,308.9	7429.1	7504.7	5373		278,808.3
Chronic care	77,469.8	92,620.5	13,163.8	2231.3	18,308.9	7429.1	4502.8	10,746		226,472.2
Outreach service	75,888.8	42,057.3	3162.7	2603.2	18,308.9		1500.9			134,212.3
MFHS	37,944.4	77,971.4	14,104.1	6800.2	8999.3	11,143.6	9005.7	5373		180,651.1
Child health	37,944.4	66,157.5	12,821.9	2656.3	18,308.9	7429.1	5253.3	2686.5		153,257.8
EPI	37,944.4	47,727.9	2051.5	2231.3	18,308.9	3651.6	1500.9	2686.5		106,793.4

^<^Exchange rate at 22.9 ETB per USD^>^

### Total annual recurrent cost

The total annual recurrent cost was USD 1,468,174.55 while the mean annual recurrent cost per health center was USD 91,760 ± 42,344.22. It also contributed to 83.7% of total annual cost. Among the recurrent cost component, the highest proportion was incurred by Personnel which accounted for USD 790,508.49 ± 16,831.01. It comprised for 53.8% of the total annual recurrent cost. The lowest proportion of recurrent cost, USD 26,562.78 was incurred by medical supplies & consumables with that also comprised for (1.9%) of the total annual recurrent cost with standard deviation of USD 794.47.

The average cost for personnel was USD 49,406.78 per health center where the greater parts (53%) were absorbed by the final cost centers followed by overhead cost center which absorbed 28% of the total personnel costs while only 19% of the total personnel cost were absorbed by intermediate cost center.

The average cost for medical supplies & consumables USD 1660.17 per health center. The total medicine, Laboratory supplies & reagents and medical supplies & consumables cost was USD 584,595.49 (28%) of total annual cost from which medicine shared USD 472,553.58 (21.9%), Laboratory supplies & reagents consumed USD 85,479.13 (4.8%) and USD 26,562.79 (1.5%) spent on medical supplies &consumables while average cost of medicine USD 29,534.61 per health center, that of Laboratory supplies & reagents was USD 5342.45 and medical supplies &consumables consumed USD 1660.17.

The average cost for medical supplies & consumables USD 1660.17 per health center. The total medicine, Laboratory supplies & reagents and medical supplies & consumables cost was USD 584,595.49 (28%) of total annual cost from which medicine shared USD 472,553.58 (21.9%), Laboratory supplies & reagents consumed USD 85,479.13 (4.8%) and USD 26,562.79 (1.5%) spent on medical supplies &consumables while average cost of medicine USD 29,534.61 per health center, that of Laboratory supplies & reagents was USD 5342.45 and medical supplies &consumables consumed USD 1660.17.

Over two third (70%) of medicines, laboratory supplies and medical consumables cost were from revolving fund where it was 74% for medicine and 52% for laboratory & medical consumables. About 100% of the total medicine, laboratory cost and 95.8% of medical consumables were allocated to the final cost centers respectively. Curative consuming the highest percentage with 25.4% of medicine, 40.5% of laboratory supplies and 30.3% medical supplies &consumables cost while emergency consume the lowest medicine cost (5.6%) and medical consumables cost (3.5%) while EPI consume the lowest laboratory supply cost (2.4%).

The Average annual cost administration and utility were USD 12,355.88 per health center with the highest cost was spent on stationary and others’ office consumables &supplies with USD 4242.67 per health center which is about 34% of the total cost of administration and utility.

**Fig. 2:**
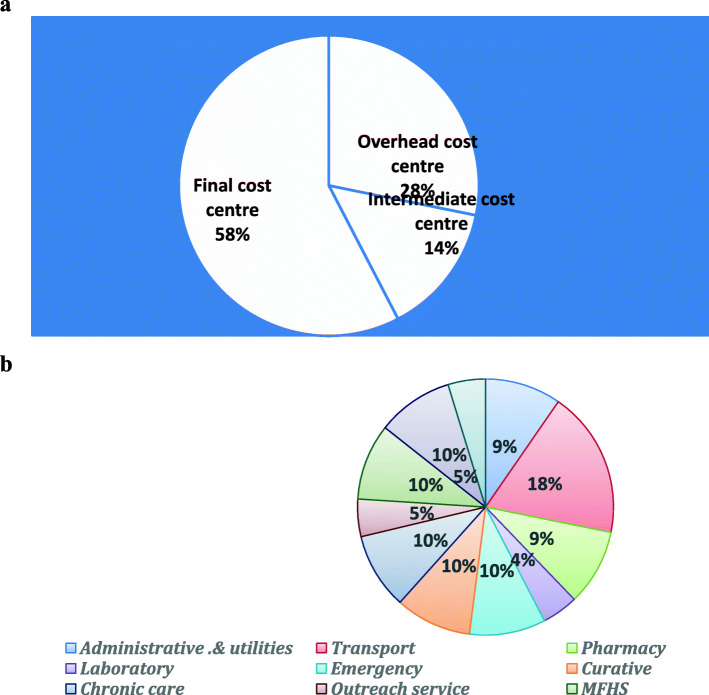
**a** Percentage of total administrative, supportive & utility cost per cost center in Jimma zone for EFY 2009, October 2018. **b** Percentage of share of total cost among each essential health services provided by PHFs in Jimma zone from final cost center for EFY 2009, October 2018

It can be evidently said that the cost center which incurred the highest cost was final cost center which consumes USD 109,542.817 for all health centers which is about 58% of the total cost of administration and utilities, followed by overhead cost center which absorbed 28% of the total administration and utility costs while only 14% of the total administration and utility cost were absorbed by intermediate cost center administration **[**Figure 2a, b. and Table 3**].**

**Table 3:** Annual Recurrent costs of delivering Essential health care services (USD) at Health centers of Jimma zone for EFY 2009, October 2018

Cost components	PHC (*n* = 16)				
	SUM	Mean	SD	% Total Annual Cost	% Recurrent Cost
Personnel	790,508.5	49,406.8	16,831.22	45%	53%
Administration	197,694.0	12,355.8	5961.32	10%	13%
Medicine	472,553.6	29,534.5	42,344.22	22%	26%
Lab.sup& cons	85,479.2	5342.4	18,762.12	5%	4.4%
Med. Sup & cons	26,562.79	1512.51	2303.18	2%	1.6%
Total recurrent cost	584,595.5	91,760.9	10,274.0	83.3%	100%

^<*^Exchange rate at 22.9 ETB per USD^>^

The total annual capital cost was USD 285,975.6 and the mean of USD 17873.4 with standard deviation of USD. It also contributed to 16.7% of total annual cost. Among the total annual capital cost the highest proportion was incurred by Health centers’ building cost with USD 75,047.28 (26.2%) & the mean annual equivalent cost of USD 4690.45 per health center followed by building with total annualized cost of USD 62958.41 & mean annual equivalent cost of USD 3934.91 per health center.

The depreciation costs of buildings accounted for USD 110,958.4 (3.6%) of the total annual costs and about 22% total annual capital cost. It can also be evidently said that the Final cost center (maternal & family health services, curative chronic, child health) have the highest annualized cost of building at USD 2745.29, as they occupied more number of rooms which consumed about 71% of the total annual buildings cost.

The depreciation of equipment also accounted for about was USD 121,104.91 (4.2%) of the total annual cost and 26.2% total annual capital cost. Half (51%) of the costs calculated under the heading “equipment” were attributable to the final cost centers while 42% to intermediate cost centers.

The total cost of furniture was USD 1679.06 per health center and accounted for 1.6% of the total health centers’ costs. Over three fourth (79%) of the costs calculated under the heading “furniture” were attributable to the final cost centers while 14% to intermediate cost centers.

The total annualized cost of the vehicles was USD 75,047.28 which represented about 6.9% of the total annual cost and about 42% of the total of the annualized capital costs. About 100% of the total vehicle cost was allocated to the overhead cost center **[**Table 4**].**

**Table 4:** Annual capital costs of delivering Essential health care services (USD) at Health centers in Jimma zone for EFY 2009, October 2018

Cost components	PHC (*n* = 16)				
	SUM	Mean	SD	% Total Annual Cost	% Recurrent Cost
Buildings	62,958.40	3934.9	1549.07	3.6%	22.01
Equipments	75,047.29	4690.4	1219.59	4.2%	26.24
Furniture’s	26,865.03	1679.1	1167.22	1.6%	9.39
Vehicles	121,104.92	7569.06	1071.88	6.9%	42.34
Total capital cost	285,975.65	17,873.4	10,274.01	16.3%	100%

^<^Exchange rate at 22.9 ETB per USD^>^

**Fig. 3:**
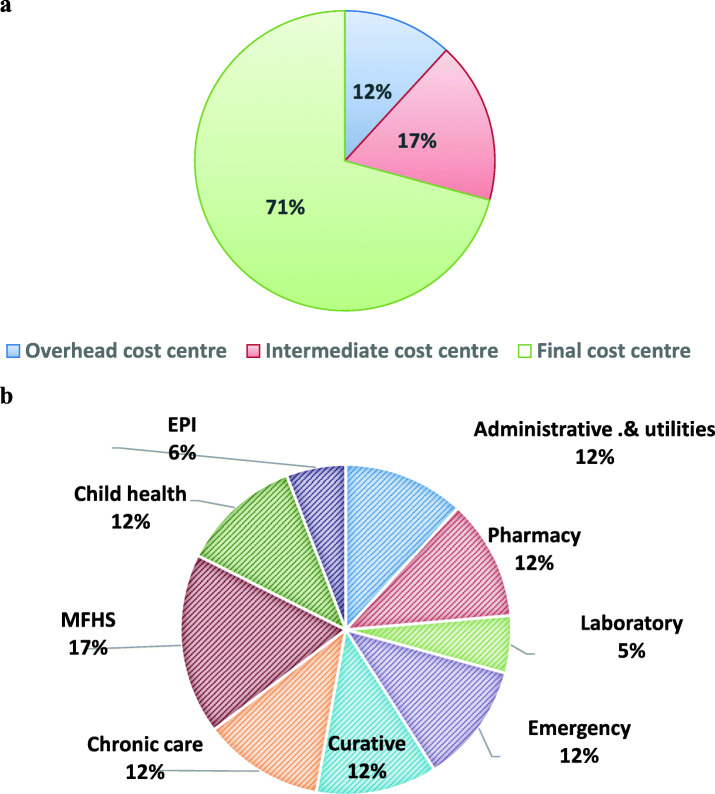
**a** Proportional allocation of buildings costs to cost centers**.** b Percentage of share of building cost among each essential health services provided by PHFs in Jimma zone from final cost center for EFY 2009, October 2018

**Fig. 4:**
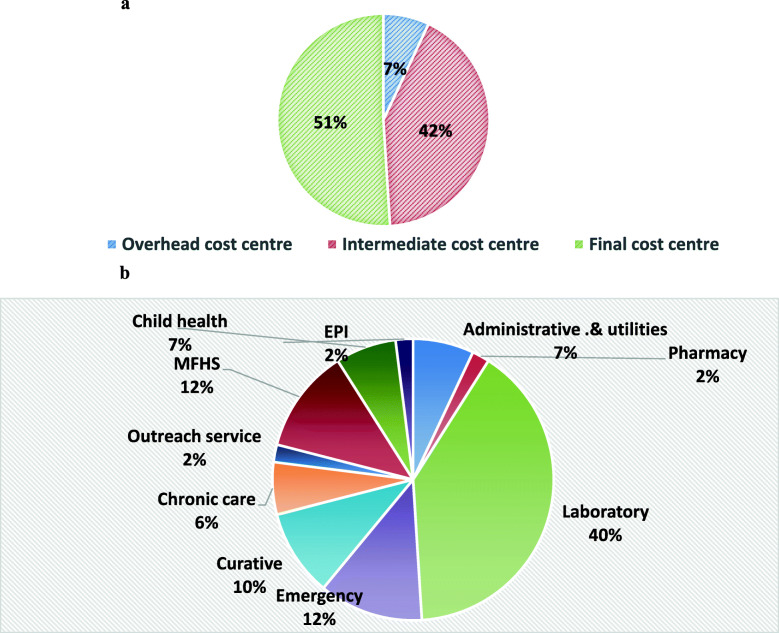
**a** Proportional allocation of equipment costs to cost centers. **b** Percentage of share of equipment cost among each essential health services provided by PHFs in Jimma zone from final cost center for EFY 2009, October 2018

The final cost center namely maternal and family health services consume more building costs compare to others that might be attributed to the number of rooms required to provide full scope of essential maternal and family health service while maternal and family health services and emergency service consume large part of furniture’s cost. Similarly, laboratory services consumed largest part of capital equipment costs that might be due number and sophistication of durable medical equipment used at the department **[**Fig. 3a, b, Fig. 4a, b**].**

### Cost of essential health services

Overall the average unit cost for all essential health services provided by the financial year was USD 7.34. The most expensive unit of output is child health service with USD 9.72 while the least expensive unit of output was being the pharmacy services USD 0.62. All health centers spent on average USD 9.72 per child health service visit, USD 9.12 per family health service visit, USD 3.84 per delivery and USD 2.79 per ANC visit. Variations in the unit cost within individual health center and between services at health centers were discovered with personnel having higher costs than other cost drivers **[**Table 5**].** The variations in the unit cost were attributable to recurrent expenditure like personnel cost drivers as capital costs (buildings, vehicle, furniture and equipments) contribute relatively small percentage (less than one fifth) to total annual cost. Hence, the resultant unit cost of services based on recurrent cost did not show much difference to that of unit cost analysis based on total cost where child health service had relatively high unit cost (USD 8.75) while pharmacy service visit (USD 0.62) had relatively low unit cost per output **[**Table 5**].**

**Table 5:** Cost of each unit of output of the EHS for cost centers (USD) in Jimma zone public health centers for EFY 2009, October 2018

Service category	Total output	Total cost	Unit cost of each service	SD
**Pharmacy**	**167,625**	104,470.96	0.62	0.17
**Laboratory**	149,283	120,581.23	0.81	0.22
**Emergency**	22,340	147,677.83	6.61	1.79
**Curative**	39,093	278,808.25	7.13	1.94
**Chronic care**	55,850	226,472.19	4.06	1.13
**Outreach service**	23,244	134,212.18	5.77	1.57
**MFH services**	97,136	180,651.09	1.86	0.50
**ANC**	29,139	81,292.98	2.79	0.75
**NC**	13,598	28,904.17	2.16	0.58
**Delivery**	15,543	59,614.85	3.84	1.04
**FP service**	1189	10,839.07	9.12	2.48
**Child health**	15,762	153,257.82	9.72	2.64
**EPI**	24,285	106,793.4	4.39	1.19
**Total**	233,264	1,452,924.90	7.34	2.12

^<^Exchange rate at 22.9 ETB per USD; unit cost is for one time visit/attendance/appointments/>

### Sensitivity analysis

The total annual costs were affected by discount rate variation and useful life of capital items at health centers to some extent but not to level of significance set prior to the study. The average annual cost of providing range of essential health services at health center varies from USD 109634.39 to USD 111682.63 on varying discount rate from 5 to 7% for the capital costs that only resulted 1.3% of change in total cost and 1.8% of change in overall all unit cost on average. Useful life of capital items affected both total capital and annual costs to some extent. For building alone using useful life 20 years instead of 30 years resulted in fluctuation of total capital cost by 22.1% (from USD 17873.47 to USD 105699.48) and affected total annual cost by 3.6% (by changing from USD7.34 to USD 7.08) on average. On the other hand, increasing the average number of the attendants at health center by 10% has lower impact on the unit costs. For instance, increasing the number of EPI beneficiaries by 10% (from 1518 to 1669) resulted in a 9.3% reduction in cost per EPI attendant (from USD 4.4 to USD 3.9) and similar reduction will happen for the child health services by increasing child health services attendants by 10%.

Effect of increasing personnel cost on total annual cost was also explored. For instance, having an expectation that the number of staff will increase in future due to government policies or health care reforms like HSTP to provide an improved health care for its’ citizen, 10% increase in personnel cost at health centers, resulted in increment of annual recurrent cost by 5.4%. Similarly, the average annual cost of providing care will increase by 4.5% (from USD 109634.3 to USD 114,575.1) while overall unit cost will increase by 4.3% (from USD 7.34 to USD 7.67).

## Discussion

Though all the study health centers had basic infrastructure listed in Ethiopian health care standards, there were variations in some of the background characteristics like the number of rooms, number and pattern of staff, the catchment areas, and the number of EHS visit during a fiscal year. Most (93.8%) of health centers had an adequate number of rooms as stated and listed in Ethiopian health center standards [36]. The average room number for health center buildings’ was nineteen, which is higher than the number stated under the minimum standard for a primary health center in Nigeria was thirteen [37]. Concerning the number of health center staff, the average number of health center staff was 21 and lower than the number stated in Ethiopian health center standards [36]. But, it is close to those recommended by a minimum standard for a primary health center in Nigeria twenty-three [37] and eighteen in India [38]. Although there were variations in the population covered by the health center, Most of the health centers were within the maximum numbers stipulated in the Ethiopian health center standards [36].

The average annualized cost for providing essential health services through a health center was USD 109,683 (USD7.4 per capita). This finding is close to finding from Syria, where average costs per EHS were USD 6.01., USD 2 per capita in Vietnam, USD 1.4 per capita in Uganda, and USD 1.3 per capita in Nepal [39]. But, it is lower than Ethiopian national average spending on health per capita of USD 20.77 in 2011/12 [40] and USD 28.65 in 2013/14 [35]. It is also lower than the findings from the study conducted in the Kassena-Nankana district of northern Ghana [14]. The difference could be due to a difference in the number of resources allocated to primary health care and the methodological difference for cost accounting.

The recurrent cost accounted for more than three fourth (83.7%) of total annual costs. In this regard, these findings close to the study done in Burkina Faso [21], Thailand [22] and Ghana [30], where recurrent cost accounted for More than three fourth (81, 76.38, and 80%) of total annual costs respectively.

The main cost drivers for health centers were personnel, drugs & consumables. Personnel cost accounted for the highest proportion, nearly half (45%) of total annual cost and 53.8% of the recurrent costs. It is close to the finding from another study done on primary health care in Ethiopia through the federal ministry of health that explained 46%of total annual costs went to human resources at health centers [6]. These findings corroborate other studies in different countries where personnel cost emerged as the highest cost component [11, 15, 18, 23, 25, 30]. However, it is higher than the figure from the Ethiopian health care delineation study, where human resource accounts for an average of 35% of the total recurrent costs [11]. The difference might be due to wage rate differentials and staffing patterns of essential health providers at health facilities. The increment of personal costs has an impact on total annual costs. It implies that personnel cost is a vital cost variable in providing essential health services that the policymakers should consider when planning on improving essential health services delivery.

Drugs & consumables were the second key cost drivers that consumed nearly one-third (29%) of the total annual cost. This finding is close to the studies done in Indonesia, Pakistan, and New Guinea. The Indonesia primary care providers spent only 27% of the total facility’s expenditure each on drugs and supplies [26], while different health facilities in Pakistan top take up to 20–30% of total recurrent cost [19]. Drugs & consumables consumed 30% annualized total cost at New Guinea [23]. However, the figure from this study is lower than the figure from two studies done in our country where finding from resource tracking and management project primary health care cost study stated, drugs & supplies constitute (40%) in health centers expenditure. Similarly, a figure from the Ethiopian health care delineation study revealed that health centers spent half (53%) of the recurrent costs of drugs & consumables on average [11]. The difference in figures between studies might be due to differences in infrastructure, supplies, drugs, and pharmaceutical logistic management performances.

Two third (65.9%) of the total annual cost was accounted in the final cost center. Curative health services accounted for the highest with 15.2%, followed by chronic care service with 12.5%, while EPI consumed the least, with 5.8% of the total annual cost per health center. The finding is contrary to the study done in 11 districts of health facilities of Ghana, where the higher percentage (66%) spent on the preventative and promotive component of essential health services [30].

Child health service had the highest unit cost (USD 9.7), which is higher than findings from Burkina Faso that indicated an average Cost per Child health as USD 27.6. Child health service followed by family health service with unit cost per output of USD 9.1, which is higher than findings from Burkina Faso that indicated an average cost per FP as USD 0.51 [21]. In contrast, the average unit cost per PNC was 2.2 USD, per laboratory service was USD 0.6, and per pharmacy service visit was USD 0.8, which were relatively low unit costs. The average unit cost per ANC visit is slightly similar to the studies in Uganda, Malawi, and Ghana, where the unit cost per ANC visit was estimated as USD 2.21 in Uganda [16], USD 3.23 in Malawi [16], and USD2.97 in Ghana [12]. Unit cost per institutional delivery is in line with the study done in Rwanda, where the unit cost per institutional delivery was USD2.71 [41].

A study done in Ethiopia on immunization of children during the Child Health Days indicated that an average cost per child per one round was USD 0.56 [22] and cost per EPI was USD 1.17 in Burkina Faso, which is lower than unit costs in this study (USD 4.4) [21]. It might be related to the lower cost of vaccination during the campaign days rather than routine vaccination activities. A possible explanation for the variation in reported unit cost might be due to the time difference between comparator studies and a possibility related to consumer prices inflation which necessitates discounting for the time difference and adjusting for inflation, even adjusting for inflation does not guarantee for valid comparison due to relatively higher inflation of health care costs as compared to the general inflation rate. In addition, location and utilization of population coverage could be reasons for the variations. Changing the discount rate for capital expenses from 5 to 7%, only resulted in 1.3% in total cost and 1.8% change in overall all unit cost, on the average change. This change is within the threshold (10%) anticipated, not much difference in annual and unit Cost with the change in discount rates and lifetimes.

### Limitations of the study

Readers should be cautious of the limitations of the study while interpreting the results. Total and unit cost estimates are affected by the quality of the available data. Record keeping at the health centers was poor and could affect the study results. Some donated items and technical assistance that provides the exempted essential health services have no cost records on them at the health centers, cost of such services were not included in the cost analysis.

Building costs was calculated indirectly based on the number of room shared among essential health services and total health center construction cost as it was difficult to obtain reliable cost of building of the health centers. Similarly, personnel cost across cost center were allocated indirectly based on the number and type of profession working at cost center rather than staff time allocation pattern that bases on time-motion to assess time contribution of staff performing multiple tasks. In spite of these stated limitations, the methodology used in this study is applicable across various settings & the estimates from this study are reflective of the current level of health centers and services delivered at them.

## Conclusions

Cost of providing essential health care service through health center is low. Recurrent cost categories and final cost centers are the major area of resource consumption at health centers. The variability in unit costs and cost components of all essential health services, suggest that potential exists to reduce costs through efficient use of both human and material resources like personnel, drugs and medical supplies. The unit costs of essential health services provided at health centers mainly depends on the intensity of use of the resources and utilization pattern for essential health services. Further work is required to explore the key drivers of efficiency and interventions that may facilitate efficiency improvements at health centers.

## Recommendations

The personnel cost was the Prime cost variable in this study. It is better if Oromiya regional health bureau and District health service managers carefully consider how much would it cost from the providers’ perspective; and whether that cost allocation affects the provision of essential health services during human resource recruitment planning on the expansion of primary health care.

The regional, and district health service managers ought to fund the health centers through proper health sector budgetary allocation, community mobilization, and seeking donor assistance for exempted services delivery since health spending is far below what is nationally recommended for public health centers to provide adequate essential health service at service delivery point.

The Oromiya regional health bureau, as the main provider, financing agency, and regulator for the district health system should intensify its efforts to control healthcare costs increment by introducing cost-control measures.

Medicines, laboratory reagents, medical supplies, and consumables were revealed in the study as one of the important cost drivers. Hence, the health center management team should manage the proper allocation to this cost category.

Antenatal care, vaccination, family planning and postnatal Care (PNC) are the main lists of the essential health service package. Health centers managing directors’ needs allocate proper amount of health centers’ resources to these final cost centers (case team) to improve provision of essential health services to population in their catchment area. Since this study was conducted from the health care provider perspective to estimate resources incurred when providing essential health services, Costs from clients’ angles and the opportunity cost of alternatives were not included in the analysis. Hence, basing this study as the starting point, future health policy and system management researchers need to explore this area using large numbers of primary health facilities in different parts to generate more generalizable results.

## Supplementary Information

**Additional file 1.**

## Data Availability

The data sets generated and/or analyzed during the present study are available from the corresponding author on reasonable request.

## Abbreviations

BPHS: Basic Package of Health Services
EHS: Essential Health Services
EHSP: Essential Health Service Package
FMOH: Federal Ministry of Health
FP: Family Planning
HC: Health Centre
HSTP: Health Sector Transformation Plan
MSH: Management Sciences for Health
PNC: Postnatal care
SDG: Sustainable Development Goal
SDP: Service Delivery Points
UHC: Universal Health Coverage
USD: United States Dollar
WDR: World Development Report
WHO: World Health Organization
