# Supplementary material for: Cost of provision of essential health Services in Public Health Centers of Jimma zone, Southwest Ethiopia; a provider perspective, the pointer for major area of public expenditure
Source: Health Econ Rev. 2021 Sep 13;11:34. doi: 10.1186/s13561-021-00334-y (PMC8436509; doi:10.1186/s13561-021-00334-y)
Supplement: Supplementary file 1 — Additional file 1. [file 13561_2021_334_MOESM1_ESM.docx]

# Supplementary file:-Annexes of sampling strategy chart, study tools, and additional data

**Annex I: - Sampling strategies followed during study in selecting the districts and the health centers for this study.**

| **JIMMA ZONE DISTRICTS** (21 districts, 118 health centers) |
| --- |

| **C-LEVEL** districts **(13D)**   \| - **O/Neda** (7 HC) - **Gera**(5 HC) - **Mecho** (6 HC) - Sigmo (5 HC) - **TiroAfeta**(6 HC) - Setama (5 HC) \| - limmu kosa(6 HC) - O/Beyem (4 HC) - **limmu seka**(6 HC) - C/ Botor (4 HC) - B/Tolay (4HC) - Gumay (3 HC) \| \| --- \| --- \| |
| --- | --- | --- |

| **A-LEVEL** districts **(3D)**   - Manna (7 HC) - **Kersa** (7HC) - Agaro town admn. (2HC) |
| --- |

| **B-LEVEL** districts **(5D)**   - **Gomma** (11 HC) - **Seka Chekorsa** (9 HC) - Dedo (8 HC) - Sokuru (6 HC) - Shabe sombo (5 HC) |
| --- |

| **SELECTED**  Districts .**(5)**   - **O/Neda** (7HC) - Sigmo (5 HC) - **Tiro Afeta**(6 HC) - Nono Benja (3 HC) - **limmu seka** (6 HC) |
| --- |

| **SELECTED**  Districts (2)   - Sokuru (6 HC) - Shabe sombo (5 HC)) |
| --- |

| **SELECTED** Districts (1)   - **Kersa** (7HC) |
| --- |

| **Selected public health centre**   - Sokuru - Baso - Shabe - Anja gambo |
| --- |

| **Selected public health centre**   - Serbo - Kara gora |
| --- |

| **Selected public health centre**   \| - Dimtu - Nono, - Soloka - Seka, - Atnago \| \| --- \|  \| - Boneya - Asendabo - Sigmo, - Robe - Ako \| \| --- \| |
| --- | --- | --- |

##

## Annex II. : - The Exhaustive list of component of essential health services given at health centers

| **Essential health service categories** | **Component of essential health services** |
| --- | --- |
| Curative/chronic care | - Emergency service ( Motor vehicle accident, gunshot and others) - Curative care (Limited disease condition) like TB, malaria, STD, ENT , ophthalmic and other notifiable disease & treatable condition) - Chronic care (ART, DM , HTN & CVD, Asthma, epilepsy) - Child health ( diarrhea, malnutrition, respiratory infection, helminthiatis, measles) |
| Preventive/ Promotive care | - Maternal and child health (maternal, child & EPI) Maternal ( FP, ANC, PNC, delivery and MWH service) - Immunization:-EPI (Vaccination) in HC - Outreach service (Nutritional and dietary consulting, BHC and health promotional service and other community health service) |

## Annex III: - Direct allocation form

| **Cost centers** | **Personnel** | **Medicines /sundries/** | **Utilities/ cleaning/** | **Building** | **Medical and non medical equipment** | **Others** | **Total** |
| --- | --- | --- | --- | --- | --- | --- | --- |
| **Overhead** |  |  |  |  |  |  |  |
| Administration |  |  |  |  |  |  |  |
| Transport |  |  |  |  |  |  |  |
| **Ancillary/intermediate** |  |  |  |  |  |  |  |
| Pharmacy |  |  |  |  |  |  |  |
| Laboratory |  |  |  |  |  |  |  |
| **Final** |  |  |  |  |  |  |  |
| Emergency |  |  |  |  |  |  |  |
| Curative |  |  |  |  |  |  |  |
| Chronic care |  |  |  |  |  |  |  |
| Outreach service |  |  |  |  |  |  |  |
| ANC |  |  |  |  |  |  |  |
| PNC |  |  |  |  |  |  |  |
| Inst. Delivery |  |  |  |  |  |  |  |
| FP |  |  |  |  |  |  |  |
| Child health |  |  |  |  |  |  |  |
| EPI |  |  |  |  |  |  |  |

## Annex IV:-Cost centers

| **Cost center** | **Allocation criteria** |
| --- | --- |
| **Overhead**  Administration, support departments and utilities  Transport | Proportion of direct costs of the cost center  Vehicle operation cost and capital vehicle/bike cost |
| **Intermediate Cost Centre**  Pharmacy  Laboratory | Value of drugs supplied to cost centers  Number of tests carried out for each cost centers |
| **Final cost center/by health service categories**  Emergency service  Curative care service  Chronic care service  Maternal and child health  ( FP, ANC/PNC, institutional delivery )  Outreach service  Child health services  EPI | No. Of Visits  HC- patient day and admission  Visit and follow up/ HC-patient  Client day /no. Of delivery and referral for higher level,  No. Of beneficiaries  No. Of beneficiaries  No. Of individual vaccinated/children +adult) |

## Annex V:-Summary of cost allocation

| **Costs** | **Method of allocation** |
| --- | --- |
| Personnel | Total personnel expenditure * %age weighted of visited |
| Administrative & utilities | Total expenditure on administrative and utilities*proportion of patients |
| Drugs | Direct (drug costs of each health centers’ calculated and allocated directly to relevant service ) |
| Lab. Supplies | Direct (drug costs of each health centers’ calculated and allocated directly to relevant service ) |
| Medical consumables | Direct (laboratory supply costs of each health centers’ calculated and allocated directly to relevant service ) |
| All drugs, laboratory reagents & Medical supplies | Direct (all drug ,lab supplies and medical consumables costs of each health centers’ calculated and allocated directly to relevant service ) |
| Buildings | Annual recurrent cost * proportion of patients |
| Vehicle | Annual recurrent cost * proportion of patients |
| Furniture | Annual recurrent cost * proportion of patients |
| Equipments | Annual recurrent cost * proportion of patients |

## Annex VI:-Cost categories

| Category Description | |
| --- | --- |
| 1. Recurrent cost | |
| Personnel cost | It includes gross income of staff, complements of earnings (i.e allowances & overtime) human resource costs and cost of performance -based incentives (both monetary and value of non-monetary). |
| Administrative cost | It includes electricity, water, telephone bills, cleaning products, repairs, post office, printing, photocopying, and stationary. In addition, cost of spare parts purchased, servicing of the means of transport, fuels and lubricants consumed were also included. |
| Pharmacy costs | These costs included the costs of drugs and vaccines consumed within the period. |
| Laboratory costs | This group included all laboratory supplies used in the period. |
| Medical supplies cost | This group included all medical consumables used in the period. |
| 1. Capital cost | |
| Cost of building | It refers to all the rooms in the health care centre. The total cost of constriction as well as Numbers of room used. |
| Vehicle costs | These included all vehicle costs (motorcycles, four-wheel vehicles, ambulance and bicycles). |
| Equipment cost | It includes cost of general equipment and equipment in the various rooms (waiting room, consulting room etc.) |
| Furniture cost | It includes cost of furniture in the various rooms (waiting room, consulting room etc.) |

##

## Annex VII:-The Exhaustive list and grouping of costs items

| **Recurrent cost** | **Personnel cost**   - all HCs’ staff Salaries - all HCs’ staff Complementary earnings | **Administrative cost**   - Telephone/fax charges - Electricity/water charges - Stationery requirements - Office materials & supplies - cleaning agents - Transport and vehicle operational cost: Tools, Spares/accessories, Fuels, lubricants, & paraffin and Servicing fees & repairs | **Pharmacy costs**   - Drugs [EML] | **Medical and Laboratory costs**   - Lab. supplies and consumables - medical supplies and consumables |
| --- | --- | --- | --- | --- |
| **Capital cost** | **Vehicles cost**   - 4- wheel - 2- wheel | **Equipments cost**   - Laboratory equipment - Office equipment - Medical equipment | **HC buildings and rooms cost** | **Furniture’s cost**   - All HC furniture like tables, file cabinet chairs |

##

## Annex-VIII:-Data collection TOOL

**Section 1: - Check lists to assess Health center characteristics**

**Section 1.1:-background information**

| Name of district | | |  |
| --- | --- | --- | --- |
| District capital | | |  |
| Health centre name | | |  |
| Years of operation/establishment | | |  |
| Location of health centre | Woreda | |  |
|  | Kebele | Urban |  |
|  |  | Rural |  |
| Catchment population | Male | |  |
|  | Female | |  |
|  | Total | |  |
| Contact number of in-charge | | |  |
| Date of assessment | | |  |

**Section 1.2:- Essential health service utilization in EFY 2009 at selected health centers**

| **Essential health services** | **Number of beneficiaries during fiscal year** |
| --- | --- |
| Emergency per year |  |
| Curative per year |  |
| Chronic care per year |  |
| ANC |  |
| PNC |  |
| Institutional Delivery |  |
| Family planning service |  |
| Child health per year |  |
| EPI per year |  |
| Outreach service per year |  |
| Others’(if any) |  |

**Section 2:- Resource inventory check list for identifying Cost of running a health center [reference year is 2009 EFY**]

**Section 2.1:-Human resource for health**

1. The number of full time staffs assigned to this health center in 2016/17 ----------

2. The salary of health centers’ staff in 2016/17 fiscal year? Total ---------- average --------

**Section 2.1.1:*-Personnel [HRH] cost at study health center***

| Code | HR by profession | **quantity** | **Gross monthly salary** | **Other benefits** (Complements of earnings | **Gross salary for the year** | **Total**  **( staff cost )** |
| --- | --- | --- | --- | --- | --- | --- |
| 1 | Administrative and supportive staffs |  |  |  |  |  |
| 2 | HO |  |  |  |  |  |
| 3 | Nurse |  |  |  |  |  |
| 4 | Midwife |  |  |  |  |  |
| 5 | Envt’tal health personnel |  |  |  |  |  |
| 6 | HE-supervisor |  |  |  |  |  |
|  | Lab. Personnel |  |  |  |  |  |
| 7 | Pharmacy personnel |  |  |  |  |  |
| 8 | Daily laborers |  |  |  |  |  |

NOTE: - Administrative staff s include staffs for worked at management , finance, HR departments while supportive staffs include janitors, guards, drivers, secretary and etc.

**Section 2.2.:-Check lists to assess Building cost**

1: In which year was this health facility built...

2: Who constructed the health facility ………..

3: What is the construction cost? (Local currency)…………………

4: Total number of rooms at health centers………

5: Activities carried out at each room………..

| **S.N.** | **Block** | **Room** | **Department using it/activities** |
| --- | --- | --- | --- |
| **1.** |  |  |  |
| **2.** |  |  |  |
| **3.** |  |  |  |
| **4.** |  |  |  |
| **5** |  |  |  |

**Section 2.3:-Check lists to assess Functioning equipment and furniture’s cost**

**Section 2.3.1*:-General and medical equipment***

|  | **Equipment** | **Number** | **Unit cost** | **Service years** | **Total** |
| --- | --- | --- | --- | --- | --- |
| **General Equipments** | Computer |  |  |  |  |
|  | Printer |  |  |  |  |
|  | Photo copy machine |  |  |  |  |
|  | Solar panel |  |  |  |  |
|  | Borehole |  |  |  |  |
|  | Generator |  |  |  |  |
|  | Pumping machine |  |  |  |  |
|  | Water tanks |  |  |  |  |
|  | **Other** |  |  |  |  |
|  |  |  |  |  |  |
| **Medical**  **Equipments** |  |  |  |  |  |
|  | Autoclave |  |  |  |  |
|  | Weighing scale |  |  |  |  |
|  | Exam couch |  |  |  |  |
|  | Blood pressure machine |  |  |  |  |
|  | **Others** |  |  |  |  |
|  |  |  |  |  |  |
|  |  |  |  |  |  |
| **Furniture’s** | Tables |  |  |  |  |
|  | Chairs |  |  |  |  |
|  | File cabinet |  |  |  |  |
|  | Others |  |  |  |  |

**Section 2.4:-Check lists to assess Transportation cost**

**Section 2.4*: - vehicles form***

| **Registration No.** | **Description/Type** | **Value/ Cost** | **Useful Life** | **Total Cost** |
| --- | --- | --- | --- | --- |
|  |  |  |  |  |
|  |  |  |  |  |
|  |  |  |  |  |

**Section 2.5:-Check lists to assess Drugs, laboratory supplies and medical consumables**

**Section 2.5.1: -Drugs and medical consumables**

| **Name Of Drug and consumables** | **Quantity consumed**  **by fiscal year** | **unit price** | **Annual Total price** |
| --- | --- | --- | --- |
| **TABS** |  |  |  |
| Artesonate-amodiaquine(6-13 years) |  |  |  |
|  |  |  |  |
| **CAPSULES** |  |  |  |
| Amoxycillin-250 |  |  |  |
| Amoxycillin-500 |  |  |  |
| Chloramphenicol |  |  |  |
|  |  |  |  |
| **INJECTIONS** |  |  |  |
| Diclofenac |  |  |  |
| Diaxepam |  |  |  |
| Gentamycine |  |  |  |
| Promethazine |  |  |  |
| Oxytocin |  |  |  |
| Buscopan |  |  |  |
|  |  |  |  |
| **SUSPENSIONS** |  |  |  |
| Amoxycillin |  |  |  |
| Co-trimoxazole |  |  |  |
| Flucloxacillin |  |  |  |
| Metronidazole |  |  |  |
| Erythromycin |  |  |  |
|  |  |  |  |
| **SYRUPS** |  |  |  |
| Paracetamol |  |  |  |
| Multivitamin |  |  |  |
| Promethazine |  |  |  |
| **OINTMENT** |  |  |  |
| diclofenac gel |  |  |  |
| Kalamin lotion |  |  |  |
| Cotton |  |  |  |
| Gauze |  |  |  |
| **Lab/med.Consumables and supplies** |  |  |  |
| Cotton |  |  |  |
| Bandage |  |  |  |
| Dispensing envelope |  |  |  |
| Paster |  |  |  |
| Gauze B. (rolls) |  |  |  |
| Surgical gloves (Box) |  |  |  |
| Plaster (rolls) |  |  |  |
| Liquid soap (Galon) |  |  |  |
| RDTs |  |  |  |
| HIV test kits |  |  |  |
| Syphilis test kits |  |  |  |
| Others |  |  |  |
|  |  |  |  |

**Section 2.6:-Check lists to assess Administrative and utility cost**

**Section 2.6.*1:- Administrative a*nd utilities costs**

| **Items** | **Direct Cost paid per that year** |
| --- | --- |
| Electricity(VRA) |  |
| Water |  |
| Telephone |  |
| Cleaning products |  |
| Repairs, plumbing, roofs etc. |  |
| Spare parts |  |
| Servicing fees |  |
| Fuel |  |
| Lubricants |  |
| **Others** |  |
| **Total costs** |  |

**Section 2.6.2:-*Stationery***

| **Name of stationery** | **Quantity consumed** | **Unit price** | **Total** | **Total value** |
| --- | --- | --- | --- | --- |
| Folders |  |  |  |  |
| Files arch |  |  |  |  |
| Flat files |  |  |  |  |
| Stapler |  |  |  |  |
| Staple pins |  |  |  |  |
| A 4 paper |  |  |  |  |
| A3 paper |  |  |  |  |
| Envelopes (A4) |  |  |  |  |
| Envelopes (A3) |  |  |  |  |
|  |  |  |  |  |
|  |  |  |  |  |
|  |  |  |  |  |

**Section 2.7:-Check lists to assess capital equipment maintenances at health centers**

| **S.N.** | **Type of capital equipment maintained** | **Frequency of maintenance** | **Unit price** | **Total** | **Total value** |
| --- | --- | --- | --- | --- | --- |
|  |  |  |  |  |  |
|  |  |  |  |  |  |
|  |  |  |  |  |  |
|  |  |  |  |  |  |
|  |  |  |  |  |  |
|  |  |  |  |  |  |
|  |  |  |  |  |  |

## Annex IX: - Additional information extracted from the study

## Distribution at health centers [ETB] in Jimma Zone [EFY 2009]

### 9.1.1. Total cost distribution at health center [Table]

| ***health centre name*** | ***Recurrent*** | | | | | | ***Capital*** | | | | ***Annual*** | | |
| --- | --- | --- | --- | --- | --- | --- | --- | --- | --- | --- | --- | --- | --- |
|  | ***personnel*** | ***Administrative and utilities*** | ***Drugs*** | ***Lab. Supp. & consu*** | ***Med.supp& cons'*** | ***Drugs, lab.&Med supp & consmbs*** | ***building*** | ***vehicles*** | ***Furniture*** | ***Equipment*** | ***Total Recurrent cost*** | ***Total capiatal cost*** | ***total annualcost*** |
| **Ako** | **1511101** | **407486** | **1072367** | **204115** | **49747** | **1326229** | **87344.06** | **333939** | **88299** | **122905.3** | **3102143** | **632487.4** | **3734630.4** |
| **A/gambo** | **759796** | **129350** | **227808** | **81596** | **23313** | **332718** | **80957.41** | **2652.01** | **25815** | **118557.1** | **1098320** | **227981.2** | **1326301.7** |
| **Asendabo** | **1436971** | **351776** | **969345** | **193112** | **72417** | **1234874** | **84712.62** | **17437.93** | **38483** | **65212.24** | **2878431** | **205845.3** | **3084276.3** |
| **Atnago** | **860848** | **124839** | **353431** | **91638** | **26182** | **471251** | **109619.4** | **2307.55** | **14420** | **49796.3** | **1326568** | **176143.1** | **1604769.4** |
| **Baso** | **702786** | **197405** | **345676** | **73207.85** | **20916.5** | **439800.4** | **81740.34** | **41150** | **11455** | **152122.4** | **1318301.5** | **286468** | **1502711.1** |
| **Boneya** | **537827** | **140830** | **221951** | **67966** | **19419** | **309336** | **121867.2** | **6189.77** | **21841** | **117219.94** | **864510** | **267117.88** | **1131627.9** |
| **Dimtu** | **1022466** | **188689** | **332409** | **97754.72** | **27929.92** | **458093.5** | **140499.9** | **17343.9** | **28258** | **98797.06** | **1545215.6** | **284898.74** | **1755513.833** |
| **K/ gora** | **934450** | **122355** | **292461** | **77529** | **42678** | **412668** | **167315.1** | **10437.82** | **76440** | **154276.91** | **1347044** | **408469.83** | **3647798.232** |
| **Nono** | **1297076** | **404069** | **1183913** | **190320** | **54856** | **1429089** | **110551.7** | **397741.9** | **55330** | **100557.88** | **2983617** | **664181.23** | **1233345.668** |
| **Robe** | **646845** | **197435** | **216755** | **49671** | **14191** | **280617** | **67348.07** | **9129.88** | **26072** | **124772.91** | **1006023** | **227322.67** | **1830114.381** |
| **Seka** | **1546321** | **463108** | **1100218** | **133626.3** | **53468** | **1287312** | **78275.16** | **405082.1** | **11844** | **111443.78** | **2955376.3** | **606645.53** | **4109526.679** |
| **Serbo** | **1349903** | **397744** | **631562** | **91477** | **18295** | **741334** | **69491.17** | **16184.15** | **33600** | **71046.98** | **2132198** | **190322.3** | **2322520.298** |
| **Shebe** | **1604639** | **407505** | **1341832** | **175630** | **50180** | **1567642** | **8672.3** | **365513.8** | **37462** | **108204. 9** | **3424776** | **519852.89** | **3562021.832** |
| **Sigimo** | **1537208** | **479785** | **1255191** | **144809** | **41374** | **1441374** | **89701.66** | **397205.9** | **96666** | **110219.03** | **3305329** | **693792.74** | **3944628.887** |
| **Sokoru** | **1580693** | **371752** | **965450** | **189356** | **66011** | **1220817** | **76322.26** | **742795.2** | **27746** | **97501.54** | **3165161.4** | **944365.28** | **3999121.742** |
| **Soloka** | **781854** | **145102** | **315975** | **96544** | **27584** | **440103** | **67977.7** | **9439.16** | **21755** | **116721.43** | **1183305** | **215893.57** | **1399198.575** |
| **Total** | **18110784** | **4529230** | **10826344** | **1958351.87** | **608561.42** | **13393257.99** | **1442396.05** | **2774550.07** | **615486** | **1719355.73** | **33636318.69** | **6551787.59** | **40188106.89** |
| **Mean** | **1159771.22** | **274605.5** | **492496.5** | **97149.36** | **34651.96** | **606292.5** | **83226.48** | **17390.91** | **28002** | **110831.4** | **1838706.82** | **285683.39** | **2511756.681** |

### 9.1.2. Total costs distribution at health centers [Figure]

- 1. **Overall unit cost at health centers**

### Unit cost of essential health services per cost centers

| ***Cost centers*** | ***Total vol.of. activities*** | ***personnel*** | ***medicine*** | ***lab.supp & consu.*** | ***Med.supp.& consu.*** | ***Adm.,supportive & utilities*** | ***Building*** | ***equipments*** | ***Furniture*** | ***Vehicle*** |
| --- | --- | --- | --- | --- | --- | --- | --- | --- | --- | --- |
| ***Adm. ,supportive & utilities*** | 215608 | 18.9 |  |  |  | 1.9 | 0.8 | 0.6 | 0.6 |  |
| ***Transport*** | 215608 |  |  |  |  | 3.7 |  |  |  | 12.8 |
| ***Pharmacy*** | 204218 | 8.5 |  |  |  | 2.1 | 0.8 | 0.2 | 0.2 |  |
| ***Laboratory*** | 149283 | 11.6 |  |  | 0.2 | 1.3 | 0.5 | 4.6 | 0.2 |  |
| ***Emergency*** | 22340 | 79.4 | 27.1 | 5.5 | 1 | 18.8 | 7.6 | 9.2 | 2.8 |  |
| ***Curative*** | 39093 | 45.4 | 70.3 | 20.3 | 4.7 | 10.7 | 4.3 | 4.4 | 3.1 |  |
| ***Chronic care*** | 55850 | 31.7 | 37.9 | 5.4 | 0.9 | 7.5 | 3.1 | 1.84 | 4.4 |  |
| ***Outreach service*** | 23244 | 74.8 | 41.4 | 3.1 | 2.5 | 8.9 |  | 1.5 |  |  |
| ***Maternal*** | 97136 | 8.94 | 18.4 | 3.3 | 1.6 | 4.3 | 2.6 | 2.1 | 1.2 |  |
| ***child health*** | 15762 | 55.15 | 96.2 | 18.6 | 3.9 | 26.6 | 10.8 | 7.6 | 3.9 |  |
| ***EPI*** | 24285 | 35.8 | 45.1 | 1.9 | 2.1 | 8.5 | 3.4 | 1.4 | 2.5 |  |
| ***Total*** | 238852 | 75.8 | 45.3 | 8.2 | 2.5 | 18.3 | 6.1 | 6.9 | 1.5 |  |

### Total annual & average unit cost per selected health center in Jimma zone

| **Health centers** | **total annual** | **total beneficiaries’** | **Overall unit cost** |
| --- | --- | --- | --- |
| **Ako** | 3734630.41 | 14561 | 256.4817258 |
| **Anja gambo** | 1326301.752 | 11892 | 111.5289062 |
| **Asendabo** | 3084276.289 | 22223 | 138.7875754 |
| **Baka gudo** | 1604769.349 | 8759 | 183.2137629 |
| **Baso** | 1502711.079 | 6727 | 223.3850274 |
| **Boneya** | 1131627.883 | 6736 | 167.9970135 |
| **Dimtu** | 1755513.833 | 11208 | 156.6304276 |
| **Kara gora** | 3647798.232 | 9514 | 383.4137305 |
| **Nono** | 1233345.668 | 22291 | 55.32931084 |
| **Robe** | 1830114.381 | 8062 | 227.0050088 |
| **Seka** | 4109526.679 | 19252 | 213.4597278 |
| **Serbo** | 2322520.298 | 33527 | 69.27313204 |
| **Shebe** | 3562021.832 | 29376 | 121.2561898 |
| **Sigimo** | 3944628.887 | 11081 | 355.9813092 |
| **Sokoru** | 3999121.742 | 14384 | 278.0257051 |
| **Soloka** | 1399198.575 | 9261 | 151.0850421 |
| **Total value** | 40188106.89 | 238854 | 168.2538575 |
| **Mean value** | 2511756.681 | 14928 | 168.253 |

### Average unit cost versus total number of output at health centers

**
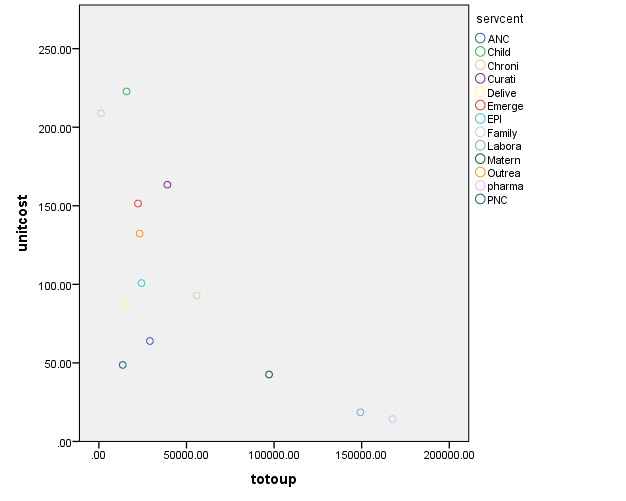
**

### Summary of cost of providing essential health services per cost center (ETB) at health centers in Jimma zone (EFY 2009)

#### Total cost

| **cost centre** | **Personnel** | **Medicine/drugs** | **Laboratory supplies** | **Med.supp. & consum.** | **Admn.& utility cost** | **Building (AEC)** | **Equipments (AEC)** | **Furniture (AEC)** | **Vehicle (AEC)** | **total Annual cost** |
| --- | --- | --- | --- | --- | --- | --- | --- | --- | --- | --- |
| **Overhead cost centre** |  |  |  |  |  |  |  |  |  |  |
| **Administrative .& utilities** | 4093037.6 |  |  |  | 419461.4 | 170202.7 | 120354.9 | 123097.1 |  | 4926153.9 |
| **Transport** | 869317.7 |  |  |  | 807830 |  |  |  | 2774550 | 4451697.7 |
| **Intermediate cost centre** |  |  |  |  |  |  |  |  |  |  |
| **Pharmacy** | 1738635.5 |  |  |  | 419461.4 | 170202.7 | 34387.1 | 30774.2 |  | 2393461.1 |
| **Laboratory** | 1738635.5 |  |  | 25559.5 | 199066.4 | 80774.2 | 687742.3 | 30774.2 |  | 2762552.2 |
| **Final cost centre** |  |  |  |  |  |  |  |  |  |  |
| **Emergency** | 1774857.1 | 606275.3 | 123376.2 | 21299.6 | 419461.4 | 170202.7 | 206322.7 | 61548.5 |  | 3383343.6 |
| **Curative** | 1774857.0 | 2749891.4 | 793132.8 | 185002.7 | 419461.4 | 170202.7 | 171935.6 | 123097.1 |  | 6387580.7 |
| **Chronic care** | 1774857.0 | 2121963.4 | 301586.3 | 51119.2 | 419461.4 | 170202.7 | 103161.3 | 246194.3 |  | 5188545.7 |
| **Outreach service** | 1738635.4 | 963544.6 | 72459.1 | 59639.1 | 206175.9 |  | 34387.1 |  |  | 3074841.2 |
| **MFHS** | 869317.7 | 1786346.8 | 323128.2 | 155791.7 | 419461.4 | 255304.1 | 206322.7 | 123097.1 |  | 4138769.7 |
| **Child health** | 869317.7 | 1515688.2 | 293752.9 | 60856.1 | 419461.4 | 170202.7 | 120354.9 | 61548.6 |  | 3511182.5 |
| **EPI** | 869317.7 | 1093460.7 | 47000.4 | 51119.2 | 206175.9 | 83658.9 | 34387.1 | 61548.6 |  | 2446668.7 |

#### Recurrent costs spent as percentage of both total annual and recurrent cost at health centers

### Capital costs spent as percentage of both total annual and capital cost at health centers

## 9.6. Sensitivity analysis [EFY 2009]

### 9.6.1. Effect of varying useful life & discounting rate on Annual cost of EHS (ETB)

| ***Variables*** | | ***capital cost*** | ***total cost*** | ***pharmacy cost*** | ***Laboratory cost*** | ***Emergency service cost*** | ***Curative service cost*** | ***Chronic care*** | ***Outreach service cost*** | ***ANC service cost*** | ***PNC service cost*** | ***Inst. Deliveryservice cost*** | ***Family PLN service cost*** | ***Child health service cost*** | ***EPI service cost*** |
| --- | --- | --- | --- | --- | --- | --- | --- | --- | --- | --- | --- | --- | --- | --- | --- |
| ***Discounting rate*** | ***5 %*** | *409486.7* | *2511756.7* | *149591.3* | *172659.5* | *211458.9* | *399223.8* | *324284.1* | *192177.6* | *116402.9* | *41387.7* | *85362.1* | *15520.4* | *219448.9* | *152916.8* |
|  | ***7 %*** | *456412.7* | *2558682.7* | *152258.2* | *175737.7* | *215228.9* | *406341.2* | *330065.5* | *195603.7* | *118467.6* | *42138.5* | *86863.8* | *15848.7* | *223361.3* | *155643* |
| ***Change in %*** | | *10.3* | *1.8* | *1.7* | *1.7* | *1.7* | *1.8* | *1.8* | *1.7* | *1.7* | *1.8* | *1.7* | *2.1* | *1.7* | *1.7* |
| ***Building*** | |  |  |  |  |  |  |  |  |  |  |  |  |  |  |
| ***Using 20 years life span*** | | *319337.1* | *2421607.1* | *135609.9* | *157404.5* | *198571.7* | *365662.7* | *297857.7* | *176777.3* | *106550.7* | *38745.7* | *77491.4* | *14529.6* | *200993.4* | *140453.2* |
| ***Percentage change (%)*** | | *22.1* | *3.6* | *9.3* | *8.8* | *6.1* | *8.4* | *8.1* | *8* | *8.5* | *6.4* | *9.2* | *6.4* | *8.4* | *8.2* |
| ***Vehicle*** | |  |  |  |  |  |  |  |  |  |  |  |  |  |  |
| ***Using 5 years life span*** | | *401151.9* | *2503421.9* | *140191.6* | *162722.4* | *205280.6* | *378016.7* | *307920.9* | *182749.8* | *110150.6* | *40054.8* | *80109.5* | *15020.5* | *207784.1* | *145198.5* |
| ***Percentage change (%)*** | | *2.1* | *0.3* | *6.4* | *5.8* | *2.9* | *5.3* | *5* | *4.9* | *5.4* | *3.2* | *6.2* | *3.2* | *5.3* | *5.1* |
| ***Furniture*** | |  |  |  |  |  |  |  |  |  |  |  |  |  |  |
| ***Using 10 years life span*** | | *400425.6* | *2502695.6* | *140150.9* | *162675.2* | *205221* | *377907* | *307831.6* | *182696.8* | *110118.6* | *40043.1* | *80086.3* | *15016.2* | *207723.7* | *145156.3* |
| ***Percentage change (%)*** | | *2.2* | *0.4* | *6.3* | *5.8* | *2.9* | *5.3* | *5.1* | *4.9* | *5.4* | *3.2* | *6.2* | *3.2* | *5.3* | *5.1* |
| ***Equipment*** | |  |  |  |  |  |  |  |  |  |  |  |  |  |  |
| ***Using 10 years life span*** | | *381278.7* | *2483548.7* | *139078.7* | *161430.7* | *203650.9* | *375015.8* | *305476.5* | *181299.1* | *109276.1* | *39736.8* | *79473.6* | *14901.3* | *206134.5* | *144045.8* |
| ***Percentage change (%)*** | | *6.9* | *1.1* | *7* | *6.5* | *3.4* | *6.1* | *5.8* | *5.7* | *6.1* | *3.9* | *6.8* | *3.9* | *6.1* | *5.8* |

### 9.6.2. Effect of varying useful life & discounting rate on unit cost on some of EHS [ETB]

| ***Variables*** | | ***pharmacy cost*** | ***Laboratory cost*** | ***Emergency service cost*** | ***Curative service cost*** | ***Chronic care*** | ***Outreach service cost*** | ***ANC service cost*** | ***PNC service cost*** | ***Inist. delivery service cost*** | ***Family PLN service cost*** | ***Child health service cost*** | ***EPI service cost*** |
| --- | --- | --- | --- | --- | --- | --- | --- | --- | --- | --- | --- | --- | --- |
| ***Discounting rate*** | ***5 %*** | 14.3 | 18.5 | 151.5 | 163.4 | 92.9 | 132.3 | 63.9 | 48.7 | 87.9 | 208.9 | 222.8 | 100.9 |
|  | ***7 %*** | 14.5 | 18.8 | 154.2 | 166.3 | 94.6 | 134.6 | 65.1 | 49.6 | 89.5 | 213.3 | 226.7 | 102.5 |
| ***Change in %*** | | 1.4 | 1.6 | 1.8 | 1.7 | 1.8 | 1.7 | 1.8 | 1.8 | 1.7 | 2.1 | 1.7 | 1.6 |
| ***Building*** | |  |  |  |  |  |  |  |  |  |  |  |  |
| ***Using 20 years life span*** | | 12.9 | 16.9 | 142.2 | 149.7 | 85.3 | 121.7 | 58.5 | 45.6 | 79.8 | 195.5 | 204.1 | 92.5 |
| ***Percentage change (%)*** | | 9.8 | 8.6 | 6.1 | 8.4 | 8.2 | 8.1 | 8.5 | 6.4 | 9.2 | 6.4 | 8.4 | 8.3 |
| ***Vehicle*** | |  |  |  |  |  |  |  |  |  |  |  |  |
| ***Using 5 years life span*** | | 13.4 | 17.4 | 147.1 | 154.7 | 88.2 | 125.8 | 60.5 | 47.1 | 82.5 | 202.1 | 210.9 | 95.7 |
| ***Percentage change (%)*** | | 6.3 | 5.9 | 2.9 | 5.3 | 5.1 | 4.9 | 5.3 | 3.3 | 6.1 | 3.3 | 5.3 | 5.2 |
| ***Furniture*** | |  |  |  |  |  |  |  |  |  |  |  |  |
| ***Using 10 years life span*** | | 13.4 | 17.4 | 146.9 | 154.7 | 88.2 | 125.8 | 60.5 | 47.1 | 82.4 | 202.1 | 210.9 | 95.6 |
| ***Percentage change (%)*** | | 6.5 | 5.8 | 2.9 | 5.3 | 5.1 | 4.9 | 5.4 | 3.3 | 6.2 | 3.2 | 5.4 | 5.2 |
| ***Equipment*** | |  |  |  |  |  |  |  |  |  |  |  |  |
| ***Using 10 years life span*** | | 13.3 | 17.3 | 145.9 | 153.5 | 87.5 | 124.8 | 60 | 46.8 | 81.8 | 200.5 | 209.2 | 94.9 |
| ***Percentage change (%)*** | | 7.3 | 6.5 | 3.7 | 6.1 | 5.8 | 5.7 | 6 | 3.9 | 6.9 | 4.1 | 6.1 | 5.9 |

### 9.6.3. Effect of increasing service volume on unit cost on some of EHS [ETB]

| ***Variables*** | ***pharmacy cost*** | ***Laboratory cost*** | ***Emergency service cost*** | ***Curative service cost*** | ***Chronic care*** | ***Outreach service cost*** | ***ANC service cost*** | ***PNC service cost*** | ***Inist. delivery service cost*** | ***Family PLN service cost*** | ***Child health service cost*** | ***EPI service cost*** |
| --- | --- | --- | --- | --- | --- | --- | --- | --- | --- | --- | --- | --- |
| Using average pharmacy service = 10477 | 14.3 |  |  |  |  |  |  |  |  |  |  |  |
| 10% increase in pharmacy service = 11524 | 12.9 |  |  |  |  |  |  |  |  |  |  |  |
| Change in percent | 9.1 |  |  |  |  |  |  |  |  |  |  |  |
| Using average laboratory visit = 9330 |  | 18.5 |  |  |  |  |  |  |  |  |  |  |
| 10% increase in laboratory visit = 10263 |  | 16.8 |  |  |  |  |  |  |  |  |  |  |
| Change in percent |  | 9.1 |  |  |  |  |  |  |  |  |  |  |
| Using average emergency visit = 1396 |  |  | 151.4 |  |  |  |  |  |  |  |  |  |
| 10% increase in emergency visit = 1536 |  |  | 137.9 |  |  |  |  |  |  |  |  |  |
| Change in percent |  |  | 9 |  |  |  |  |  |  |  |  |  |
| Using average curative care visit = 2443 |  |  |  | 163.4 |  |  |  |  |  |  |  |  |
| 10% increase in curative care visit = 2688 |  |  |  | 148.5 |  |  |  |  |  |  |  |  |
| Change in percent= |  |  |  | 8.9 |  |  |  |  |  |  |  |  |
| Using average chronic care visit = 3491 |  |  |  |  | 92.9 |  |  |  |  |  |  |  |
| 10% increase in chronic care visit = 3840 |  |  |  |  | 84.5 |  |  |  |  |  |  |  |
| Change in percent |  |  |  |  | 9. |  |  |  |  |  |  |  |
| Using average outreach beneficiaries’ =1453 |  |  |  |  |  | 132.2 |  |  |  |  |  |  |
| 10% increase in outreach service visit = 1598 |  |  |  |  |  | 120.2 |  |  |  |  |  |  |
| Change in percent= |  |  |  |  |  | 9.1 |  |  |  |  |  |  |
| Using average ANC visit = 1821 |  |  |  |  |  |  | 63.9 |  |  |  |  |  |
| 10% increase in ANC visit =2003 |  |  |  |  |  |  | 58.1 |  |  |  |  |  |
| Change in percent |  |  |  |  |  |  | 9.3 |  |  |  |  |  |
